# Different strategies for mechanical VENTilation during CardioPulmonary Bypass (CPBVENT 2014): study protocol for a randomized controlled trial

**DOI:** 10.1186/s13063-017-2008-2

**Published:** 2017-06-07

**Authors:** Elena Bignami, Marcello Guarnieri, Francesco Saglietti, Enivarco Massimo Maglioni, Sabino Scolletta, Stefano Romagnoli, Stefano De Paulis, Gianluca Paternoster, Cinzia Trumello, Roberta Meroni, Antonio Scognamiglio, Alessandro Maria Budillon, Vincenzo Pota, Alberto Zangrillo, Ottavio Alfieri

**Affiliations:** 1 Department of Anaesthesia and Intensive Care, IRCCS San Raffaele Scientific Institute, Via Olgettina 60, 20132 Milan, Italy; 2 Department of Anaesthesia, Intensive Care and Medical Biotechnologies University of Siena, Siena, Italy; 3 Department of Anaesthesiology and Intensive Care, Azienda Ospedaliera Universitaria Careggi, Florence, Italy; 4 Department of Cardiovascular Sciences, Catholic University of the Sacred Heart, 00168 Rome, Italy; 5 Department of Anesthesia and Intensive Care, Pineta Grande Private Hospital, 80122 Castelvolturno, Italy; 6 Department of Cardiac Surgery, IRCCS San Raffaele Scientific Institute, Milan, Italy; 7 Department of Cardiac Surgery, Parma University Hospital, Parma, Italy; 8 Department of Cardiovascular Anaesthesia and Intensive Care, Azienda Ospedaliera S. Carlo, Potenza, Italy; 9 Section of Anesthesia and Intensive Care, Department of Neurosciences, Reproductive and Odontostomatological Sciences, University of Naples “Federico II”, Via Pansini 16, Naples, Italy

**Keywords:** Protective ventilation, Cardiopulmonary bypass, Respiratory insufficiency, Low tidal volume, CPAP, Postoperative pulmonary complications, Systemic inflammatory response

## Abstract

**Background:**

There is no consensus on which lung-protective strategies should be used in cardiac surgery patients. Sparse and small randomized clinical and animal trials suggest that maintaining mechanical ventilation during cardiopulmonary bypass is protective on the lungs. Unfortunately, such evidence is weak as it comes from surrogate and minor clinical endpoints mainly limited to elective coronary surgery. According to the available data in the academic literature, an unquestionable standardized strategy of lung protection during cardiopulmonary bypass cannot be recommended. The purpose of the CPBVENT study is to investigate the effectiveness of different strategies of mechanical ventilation during cardiopulmonary bypass on postoperative pulmonary function and complications.

**Methods/design:**

The CPBVENT study is a single-blind, multicenter, randomized controlled trial. We are going to enroll 870 patients undergoing elective cardiac surgery with planned use of cardiopulmonary bypass. Patients will be randomized into three groups: (1) no mechanical ventilation during cardiopulmonary bypass, (2) continuous positive airway pressure of 5 cmH_2_O during cardiopulmonary bypass, (3) respiratory rate of 5 acts/min with a tidal volume of 2–3 ml/Kg of ideal body weight and positive end-expiratory pressure of 3–5 cmH_2_O during cardiopulmonary bypass. The primary endpoint will be the incidence of a PaO_2_/FiO_2_ ratio <200 until the time of discharge from the intensive care unit. The secondary endpoints will be the incidence of postoperative pulmonary complications and 30-day mortality. Patients will be followed-up for 12 months after the date of randomization.

**Discussion:**

The CPBVENT trial will establish whether, and how, different ventilator strategies during cardiopulmonary bypass will have an impact on postoperative pulmonary complications and outcomes of patients undergoing cardiac surgery.

**Trial registration:**

ClinicalTrials.gov, ID: NCT02090205. Registered on 8 March 2014.

**Electronic supplementary material:**

The online version of this article (doi:10.1186/s13063-017-2008-2) contains supplementary material, which is available to authorized users.

## Background

Respiratory failure (RF) is a common complication in cardiac surgery, with a global incidence of 20–25%. Its clinical manifestation ranges from a mild form of respiratory failure up to an acute respiratory distress syndrome (ARDS) requiring prolonged mechanical ventilation (MV) and intensive care unit (ICU) stay [1].

The pathophysiological mechanism of RF is quite complex, but it is known that cardiopulmonary bypass (CPB) plays a major role in determining lung injury [2]. A number of factors contribute to this injury: atelectasis, hyperoxygenation causing the release of free radicals [3] and a CPB-related systemic inflammatory response [4, 5].

It is a common practice to suspend ventilation during CPB, since lung function is carried out by an extracorporeal gas exchanger. However, the interruption of MV during CPB is associated with the development of micro-atelectasis, hydrostatic pulmonary edema, reduced lung compliance and surfactant diffusion.

A recent observational study [5] has identified the duration of CPB as an important risk factor for the development of microbiologically documented pneumonia.

Over recent years, several preventive lung-protective strategies have been investigated and proposed [6, 7]: ultrafiltration to remove neutrophils [8], controlled hemodilution (with a hematocrit higher than 23%), steroids [9] and adjusting MV settings during CPB, such as the application of positive end-expiratory pressure (PEEP) or continuous positive airway pressure (CPAP) of 5–15 cmH_2_O, low tidal-high frequency ventilation (100 acts/min), the application of 100% oxygen inspired fraction (FiO_2_), and bilateral CPB which involves using the lungs for blood oxygenation [10].

A recent meta-analysis [11] based on 16 clinical trials found an increase in oxygenation and a reduction in shunt fraction [11, 12] immediately after weaning from CPB if CPAP was applied during CPB. Similar results were obtained with a lung recruitment maneuver (RM) [13] at the end of CPB. Furthermore, maintaining MV during the entire duration of extracorporeal circulation would reduce the CPB-related inflammatory response and resultant tissue damage [14–16]. Unfortunately, although adequately planned, studies are not powered enough to recommend maintaining MV during CPB as an evidence-based strategy to prevent respiratory complications because major indicators of clinical outcome (i.e., duration of postoperative MV, length of ICU and hospital stay, and long-term follow-up) have not been investigated [17–19]. Therefore, according to the available data in the academic literature, an unquestionable standardized strategy of lung protection during CPB cannot be recommended [20–24].

### Objectives

We designed a randomized controlled trial to investigate the effects of three different ventilator strategies in the short, medium and long term. We are testing the hypothesis that MV during CPB would reduce the incidence of RF, defined as an arterial oxygen tension (PaO_2_) and inspiratory oxygen fraction (FiO_2_) ratio (PaO_2_/FiO_2_) <200, and other postoperative pulmonary complications (PPCs).

We identified the PaO_2_/FiO_2_ ratio as a summarizing parameter, even if it is still imprecise and unspecific, because many different conditions leading to respiratory insufficiency and hypoxemia can be assessed as a PaO_2_/FiO_2_ ratio modification. We chose it because we were looking for an objective value that can be easily calculated by any trained health care operator and that is not affected by the physician’s judgment. A decreased lung compliance, increased alveolo-arterial oxygen difference and increased intrapulmonary shunt fraction may all lead to a decreased (PaO_2_/FiO_2_) ratio. [25, 26]

## Methods/design

### Trial design

**Fig. 1:**
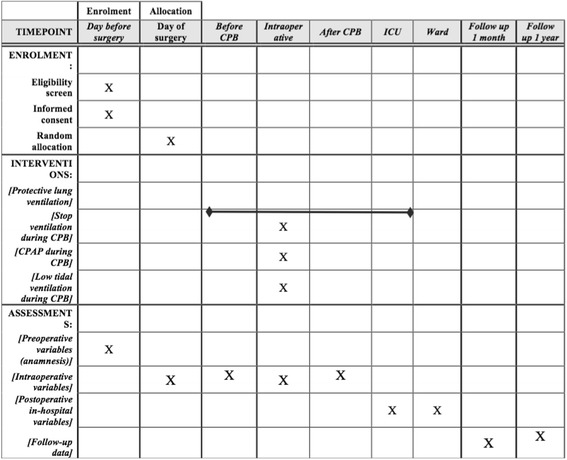
The SPIRIT figure of this trial

The CPBVENT study is a nonpharmacological, multicenter, single-blind, randomized controlled trial. Protocol structure was written in compliance to the Consolidated Standards of Reporting Trials (CONSORT) 2010 Statement guidelines and follows the Standard Protocol Items: Recommendations for Interventional Trials (SPIRIT) Statement). The SPIRIT checklist of this trial can be found in Additional file 1. The SPIRIT figure of this trial is illustrated in Fig. 1.

The study has been registered at ClinicalTrials.gov with the registration number NCT02090205 and was endorsed by the Study Group on Cardiothoracic and Vascular Anesthesia of the Italian Society of Anesthesia and Intensive Care Medicine (SIAARTI).

### Participants

After Ethics Committee approval, on 2 October 2014 (Approval number 69/INT/2014), we intend to enroll patients aged 18 years or over who are undergoing elective cardiac surgery with planned use of CPB, aortic cross-clamping, median sternotomy and two-lung ventilation. All patients will provide written informed consent before their inclusion in the trial. The inclusion and exclusion criteria are shown in Table 1 (LAS VEGAS study: NCT01601223, [27]).

**Table 1:** Inclusion/exclusion criteria

Eligibility criteria	
Inclusion criteria • Age ≥18 years • Ability to provide informed consent • Elective cardiac surgery • Surgical intervention performed with CPB, aortic cross-clamping and cardioplegic arrest • Valvular surgery, coronary surgery, surgery on the ascending aorta, combined cardiac surgery • Median sternotomy and bi-pulmonary ventilation	Exclusion criteria • Patient’s refusal • Nonelective cardiac surgery • Previous cardiac surgery • Anticipated circulatory arrest, aortic endoprothesis, TAVI, Mitraclip • Thoracotomic approach, with one lung ventilation • Patients with BMI >30 [24] • Patients with end-stage chronic kidney disease (defined as need for dialysis) • Patients with known respiratory diseases (ongoing respiratory infections, asthma, COPD, OSAS) • Patients already intubated before arrival in operating theatre • Pneumonia in the previous 30 days • Previous pulmonary resection • Patients with a preoperative oxygen saturation <90%, or P_a_O_2_ < 60 mmHg without supplemental oxygen, or a PaO_2_/FiO_2_ ratio <300, or P_a_CO_2_ > 45 mmHg • Patients with hepatic disease, defined as elevated liver enzymes (higher than two reference intervals) • Patients with pulmonary hypertension (defined as preoperative estimated systolic pulmonary artery pressure >45 mmHg)

*BMI* Body Mass Index, *CPB* cardiopulmonary bypass, *COPD* chronic obstructive pulmonary disease, *FEV*_*1*_ forced expiratory volume in 1 s, *FiO*_*2*_ fraction of inspired oxygen, *HF* high-frequency, *OSAS* obstructive sleep apnea syndrome, *PaCO*_*2*_ arterial partial carbon dioxide pressure, *PaO*_*2*_ arterial partial oxygen pressure, *TAVI* transcatheter aortic valve implantation

### Endpoints

The primary endpoint will be the incidence reduction of PaO_2_/FiO_2_ ratio <200 until discharge from the ICU [28, 29].

The secondary endpoints will be the evaluation of the following:

- Readmission to the ICU for RF
- The global incidence of PPCs after cardiac surgery (see Table 2 for complete definition)

**Table 2:** Definition of postoperative pulmonary complications (PPCs)

Complication	Definition
Respiratory insufficiency	At least one of the following criteria: • SpO_2_ < 90% • PaO_2_/FiO_2_ < 300 • PaCO_2_ > 45 mmHg • Dyspnea with respiratory distress or use of accessory muscles
Respiratory infection	Evidence of new pulmonary infiltrates on chest radiograph plus at least two of the following Johanson criteria: • Body temperature >38 °C or <35.5 °C • White blood cell count >12,000 mm^3^ or <4000 mm^3^ • Purulent sputum • Presence of a new or evolving infiltrate on chest radiograph within 7 days after surgery
Pleural effusion	Evidence of new hazy opacity of one hemithorax with preserved vascular shadows on the supine radiograph, or posterior costophrenic angle blunting on a lateral chest radiograph, or evidence of a new hypo-anechoic area between visceral and parietal pleura on chest ultrasonography
Atelectasis	Evidence on chest radiography of new parenchymal thickening surrounded by hyperinflated lung
Aspiration pneumonitis	Inhalation of gastric content in the perioperative period with subsequent acute lung injury
Bronchospasm	New expiratory wheezing responsive to treatment with bronchodilators
Pneumothorax	Presence of air within the pleural space detected with chest radiograph or loss of lung sliding of gliding sign on ultrasonographic lung examination

*PaO2* arterial partial oxygen pressure, *PaCO2* arterial partial carbon dioxide pressure, *SpO*_*2*_ oxygen saturation
- Need for re-intubation
- Need for noninvasive ventilation
- Duration of mechanical ventilation
- Length of the ICU and hospital stay
- Cardiovascular complications
- Short-term and long-term mortality
- Postoperative infections
- Postoperative residual curarization (PORC): measured with a Train of Four (TOF) and defined as a “need for pharmacological reversal”

### Interventions (randomization and treatment protocol)

The randomization list was created by the coordinating center with a dedicated software and stratified per center, in a 1:1:1 ratio, in blocks of 30. Once the patient has provided informed consent, the investigator logs into a dedicated on-line portal to obtain the allocation arm. From that moment it will be impossible to remove the patient’s record card from the online platform and, in any case, the patient will be analyzed according to the intention-to-treat principle. Any deviation from the ventilation protocol, together with reason for deviation, will be recorded on the Case Report Form (CRF). All patients will be kept blind to the allocation.

**Fig. 2:**
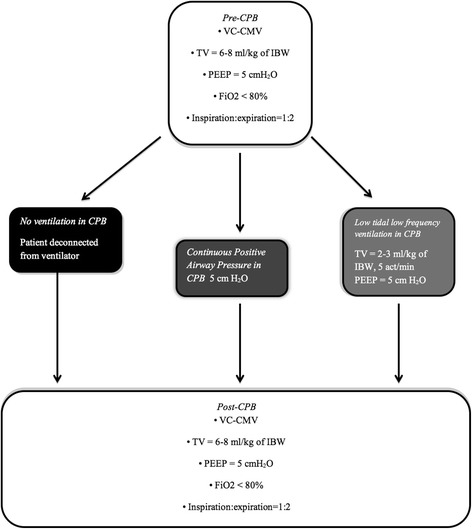
Ventilation flowchart. Description of the ventilatory strategies used before, during and after cardiopulmonary bypass. Abbreviations: *CPB* cardiopulmonary bypass, *VC-CMV* volume-controlled continuous mandatory ventilation, *TV* tidal volume, *IBW* ideal body weight, *PEEP* positive end-expiratory pressure, *FiO*_*2*_ inspired oxygen fraction

Patients will be randomly assigned to receive one of the following ventilator strategies [20] (Fig. 2):

- *First arm*. No mechanical ventilation during CPB: patient will be disconnected from the respiratory circuit
- *Second arm*. Patients will receive CPAP with PEEP of 5 mmH_2_O and FiO_2_ < 80%. To perform CPAP the ventilator will be set in manual/spontaneous mode, with a flow of 1–2 L/min and the adjustable pressure valve (APL) set at 5 cmH_2_O. The actual pressure will be checked with a pressure gauge integrated in the ventilator and a pressure gauge connected to the proximal end of the endotracheal tube
- *Third arm*. Patients will be ventilated with a respiratory rate of 5 acts/min, with tidal volume (TV) of 2–3 mL/Kg of ideal body weight (IBW) and PEEP of 3–5 cmH_2_O

Before and after CPB patients will receive a lung-protective ventilator strategy [23, 30–32], with a volume-controlled continuous mandatory ventilation (VC-CMV) mode along with the following parameters [20] (Fig. 2):

- Tidal volume (TV) = 6–8 ml/Kg of IBW [22]
- PEEP = 5 cmH_2_O
- FiO_2_ < 80%
- I:E = 1:2 (inspiration:expiration ratio)

During CPB our goal will be to maintain PaO_2_ between 200 and 250 mmHg in order to avoid hyperoxia-induced lung injury [3, 33]; moreover, the hematocrit will be maintained above 24% [34]. During weaning from CPB we will perform a single alveolar RM. This RM will be performed manually by the anesthesiologist with a gas mixture of oxygen and air (with an inspired oxygen fraction lower than 80%), keeping an airway pressure of 40 cmH_2_O for at least 7 s [35]. RM duration and any additional RM in ICU will be recorded. Immediately after the end of RM, ventilation with PEEP will be resumed. If any additional RM is be performed in the ICU, this will be done with the same procedure as in the operating room.

A FiO_2_ lower than 80% will be set during all perioperative time, since a higher oxygen fraction is widely recognized as harmful [3, 21, 36]. The oxidative stress caused by hyperoxia could itself be a source of lung damage.

### Postoperative ventilation

The anesthesiologist will report in the CRF the mechanical ventilation setting used during patient transfer from the operating theatre to the ICU. In the ICU we will apply a VC-CMV with the same parameters used in the operating room. Blood oxygen saturation will be constantly monitored with a pulse oximeter. We will report the extubation time, the duration of mechanical ventilation and the need for re-intubation. Blood gas analyses will be performed by the clinician according to clinical needs.

### Perioperative management and monitoring

All participating patients, regardless of the study arm into which they are randomized, will be monitored and managed following general standard of care practices which aim at maintaining optimal conditions. Both intraoperative and immediate postoperative anesthetic management (unrelated to ventilatory management) will be decided by the attending physician as they see fit, following the established protocols at each center. Any decision affecting the protocol will be recorded on the electronic CRF (eCRF).

Intraoperative monitoring will include an electrocardiogram (ECG), pulse oximeter, capnography, urine output, invasive blood pressure measurements, advanced hemodynamic monitoring (pulmonary artery catheter (PAC)/transesophageal echocardiogram (TEE)), bladder or esophageal temperature, and Activated Clotting Time (ACT). Monitoring of anesthetic depth analysis (bispectral analysis, BIS) and neuromuscular blockade (with TOF) are optional depending on the standard clinical practice and availability of equipment at each hospital.

The anticoagulant protocol is as follows: heparin (3 mg/Kg) to achieve an ACT of 200 s for cannulation and 480 s to proceed with the CPB. At the end of the CPB, protamine (3 mg/Kg) is used (ACT target <150). In case of allergy to heparin, we will administer bivalirudin.

During CPB, mild hypothermia (31–33 °C) and pump flow of 2.5 L/min/m^2^ will be applied.

Ventilatory parameters will be monitored by the anesthesia machine: TV, PEEP, FiO_2_, peak airway pressure (Paw) and plateau pressure (Pplat).

### Data collection and follow-up variables

Investigators will collect all the data on the dedicated CRF and will insert all the information required in the online platform. The coordinating center will directly receive all the information in a very simple data flow, with safe mechanisms for the protection of personal clinical information. The website uses an https format and all patients’ data will be collected anonymously. We have also implemented regular backups in order to minimize the risk of data corruption. Only the principal investigator and the data managers of the coordinating center will have access to the main database.

Data collection will include: preoperative information (anamnesis, physical examination, cardiac and pulmonary function, laboratory analysis), intraoperative data (ventilatory parameters, type of anesthesia, type of cardioplegia, type of CPB circuit, temperature during CPB, use of volatile anesthetics in CPB, volume and type of fluids administered, transfusion requirements, use of vasoactive drugs, duration of intervention, ventilation mode used during transport to the ICU), postoperative data (use of inotropic or vasoactive drugs, mechanical devices, time to extubation, need for respiratory support or re-intubation and hospital stay). Furthermore, we will calculate the Euroscore I–II, the ACEF score [37] and the ARISCAT risk score [38].

After discharge from the hospital, patients will be phoned for the follow-up. We will record any re-admission to hospital or exitus. Follow-up will be performed 30 days, 60 days and 1 year after randomization; we will consider overall mortality.

### Statistical considerations

#### Sample size

Sample-size calculation was based on a two-sided alpha error of 0.05 and a 80% power (beta). On the basis of respiratory insufficiency incidence after cardiac surgery, we anticipate that 25% of patients will have a PaO_2_/FiO_2_ ratio <200. We expect a reduction of 35% in the incidence of this parameter. We calculate that we will need a sample size of 263 patients per group, 789 in total. Including a dropout fraction of 10%, we calculate that we will need 870 patients to complete the trial.

A significant blind interim analysis (*P* < 0.0001) will be performed once half of the patients have been recruited to assess the recruiting progress and verify that the hypotheses assumed in the calculation of the sample size are correct.

#### Data analysis

We will analyze patients in the treatment group to which they are allocated. Data will be analyzed with professional statistical software. Data will be analyzed according to the intention-to-treat principle and following a pre-established analysis plan. Dichotomous variables will be compared with the two-tailed *Χ* ^2^ test, using the Yates correction when appropriate. Continuous variables will be compared by analysis of variance or the nonparametric Kruskal-Wallis test, when appropriate. Relative risks with 95% confidence intervals, and differences between medians with 95% confidence intervals (using the Hodges-Lehmann estimation), will be calculated when appropriate. Two-sided significance tests will be used.

The major comparison will be between the two groups undergoing MV during CPB and the group with no MV.

#### Subgroup analyses

We will infer a subgroup effect if the interaction term of treatment and subgroup is statistically significant at *P* < 0.05.

### Trial organization

The IRCCS San Raffaele Scientific Institute is the coordinating center for this study and is primarily responsible for the organization of the trial, development of the randomization scheme, study database, data consistency checks, data analysis and coordination of the study centers.

As the coordinating center we created an online platform (http://www.cpbvent.it) where investigators can electronically randomize the patient and load data in the online CRF. We also assist the other centers with administrative procedures as well as during the first cases.

All parts of the study will be conducted according to the Good Clinical Practice (GCP) Statement as well as Italian and international law on clinical research [39–41].

The trial structure includes the most important Italian adult cardiac surgical centers with experience of trials under GCP.

Safety monitoring activities performed by an independent monitoring body include reviewing the protocol with emphasis on data integrity as well as participant risk and safety issues, monitoring adverse events, and ensuring that practices are in place to safeguard the confidentiality of the data and results. The monitoring body must be separate and independent from the clinical staff or anyone responsible for patient care.

The monitoring body should not have scientific, financial or other conflicts of interest related to the trial. Current or past collaborators or associates of the principal investigator should not be a part of the monitoring body.

## Discussion

Postoperative pulmonary complications are frequent after cardiac surgery. Many factors may contribute to their development, in particular CPB. Most of these complications are not severe, but when a severe complication does occur, a patient’s life may be significantly threatened. Our effort in this trial is concentrated in particular on RF (see Table 2) which is still a major cause of mortality in cardiac surgery.

### Pathophysiology of CPB-related lung injury

A possible classification of CPB-mediated lung injury may follow the anatomical structures involved. Apostolakis et al. [42] reviewed the pathological alterations in the lungs after CPB. Such histological findings may contribute to hypoxemia, ventilation/perfusion ratio (V/Q) derangements and atelectasis. In fact, an important hypothesis regarding post-CPB lung dysfunction is related to an inflammatory reaction which has been linked to the use of the CPB circuit.

Many pathophysiological steps may be involved in lung injury. Pulmonary atelectasis is but one modifiable component of pulmonary dysfunction after CPB. Apnea during CPB has been suggested to promote the activation of lysosomal enzymes in the pulmonary circulation which in turn are correlated with the incidence of postoperative pulmonary dysfunction [43–46]. CPB duration has also been associated with lung injury and mortality. Ventilation-associated pneumonia (VAP) also showed a relation with CPB time and with preoperative pulmonary conditions [47, 48].

### Feasibility and safety of mechanical ventilation during CPB

Many clinical and preclinical trials have demonstrated the feasibility and safety of MV during CPB and introduced the key question of whether ventilating the patient’s lungs during CPB can have an impact on respiratory outcome. However, these are limited to the perioperative period and do not investigate enough clinically relevant endpoints. Nevertheless, these results showed the potential effectiveness of mechanical ventilation during CPB to prevent postoperative pulmonary complications (PPCs). Although it is currently unfeasible to recommend a unique strategy of lung preservation during CPB, the growing opinion is that keeping the lung ventilated, rather than interrupting ventilation during CPB, would improve respiratory outcome.

Schreiber et al. [11] analyzed different strategies to prevent CPB-related lung injury, finding an increase in oxygenation and a reduction in shunt fraction immediately after CPB when CPAP was applied during CPB. The application of low tidal/low frequency ventilation or vital capacity maneuvers during or after CPB was also helpful in order to prevent lung injury. Nevertheless, they found a rather limited impact on postoperative clinical outcomes. However, we find these results with surrogate endpoints [11], encouraging the investigation of the real clinical impact of ventilation during CPB.

In order to minimize potential biases, we decided to keep a rigorous scheme of mechanical ventilation for the patients included in our study. In particular, ventilation with higher tidal volumes has been associated with worse respiratory outcome in cardiac surgery [27–29].

Before planning our trial, we reviewed all the randomized clinical trials on ventilation during CPB in elective adult cardiac surgery, published from 2000 to the present year [11, 49–63]. All randomized trials take into consideration small-sized samples and limited setting.

### Limitations

The CPBVENT trial will enroll patients undergoing elective cardiac surgery with CPB. Eligible patients usually have a preserved cardiac function and no significant risk factors for postoperative respiratory insufficiency [26, 27]. Our intention is to eliminate the possible selection biases and build a more homogeneous sample. This could be a limitation, since the inclusion of higher-risk patients might be the basis of a higher-powered trial, but we believe that a “clean” experimental setting would make it easier to eliminate confounding factors and the final results will be more extendible to the majority of our patients. Furthermore, eligible patients will be all cardiac surgery patients undergoing median sternotomy and CPB, including patients undergoing mammary artery bypass graft surgery. We are aware that this could be a limitation, because ventilation during mammary artery isolation may make surgery more difficult.

Other possible limitations of our trial could be the constant PEEP level and short duration of recruitment maneuvers used. A longer recruitment maneuver could significantly impair hemodynamic stability because of the increase in intrathoracic pressure in a delicate phase of surgery such as weaning from CPB.

We also acknowledge that the primary endpoint ratio that we chose is very wide and could include many different conditions. A possible alternative endpoint would have been the incidence of PPCs in the study population. On the other hand, some false positives might occur if we consider PPCs after cardiac surgery. For instance, pneumothorax is much more frequent than in other surgical settings since all patients undergo central venous catheterization and the surgery involved might include incision of the pleural surfaces. Moreover, pleural effusion and pulmonary edema might also be consequences of depressed cardiac function, with well-functioning lungs. Moreover, we will collect all data about lung complications after surgery and provide detailed etiologies for gas exchange problems in our final results.

Furthermore, we acknowledge that the incidence of 25% for a PaO_2_/FiO_2_ ratio <200 is high and, even if it is the one we experienced in our daily practice, the risk of a final underpowered result is still present. Our choice was driven by our clinical experience but also by feasibility. On the other hand, this study is still the largest ever performed on this topic and we hope to provide some important insights into CPB-related lung injury pathophysiology and prevention.

Pulmonary artery perfusion during CPB [63] and pulsatile pulmonary perfusion [64] have been proposed as a further approach to attenuate the CPB-related lung dysfunction. Still, the currently available techniques of pulmonary perfusion during CPB are still emerging and their feasibility is strongly linked to the surgeon’s experience.

Finally, we are aware that the results obtained from a specific subgroup of patients, with strict inclusion criteria, may not be generalizable to the whole population. However, since this is the first large trial ever performed on this topic, we decided to create a clean experimental setting. Further research will be necessary to assess whether our findings are extendible to other subgroups and to the general population of patients undergoing to cardiac surgery with CPB.

## Current trial status

Approval of the final protocol by the local committee at each center was obtained before starting recruitment. Patient recruitment at the coordinating center began in November 2014. As of 21 April 2017, 559 patients have been recruited into the CPBVENT trial. We plan to involve at least 20 of the most important Italian cardiac surgery centers. We expect to complete recruitment by June 2018 with results on the 1-year outcomes being available in 2019. The final results will be published as soon as the analysis has been completed.

In conclusion, after closing the present trial we hope to be able to recommend (or not) an easy and safe, evidence-based, lung-preservation strategy during CPB to all patients undergoing cardiac surgery.

## Additional file

Additional file 1: The SPIRIT checklist of this trial. (PDF 106 kb)
